# Validation of 4D flow cardiovascular magnetic resonance in TIPS stent grafts using a 3D-printed flow phantom

**DOI:** 10.1186/s12968-023-00920-5

**Published:** 2023-02-13

**Authors:** Christoph Riedel, Inka Ristow, Alexander Lenz, Bjoern P. Schoennagel, Marko Hoffmann, Felix Piecha, Gerhard Adam, Scott B. Reeder, Peter Bannas

**Affiliations:** 1 Department of Diagnostic and Interventional Radiology and Nuclear Medicine, University Medical Center Hamburg-Eppendorf, Martinistrasse 52, 20246 Hamburg, Germany; 2 Institute of Multiphase Flows, Hamburg University of Technology, Hamburg, Germany; 3 I. Department of Medicine, University Medical Center Hamburg-Eppendorf, Hamburg, Germany; 4 Department of Radiology, University of Wisconsin-Madison, Madison, WI USA; 5 Department of Medical Physics, University of Wisconsin-Madison, Madison, WI USA; 6 Department of Biomedical Engineering, University of Wisconsin-Madison, Madison, WI USA; 7 Department of Medicine, University of Wisconsin-Madison, Madison, WI USA; 8 Department of Emergency Medicine, University of Wisconsin-Madison, Madison, WI USA

**Keywords:** Transjugular intrahepatic portosystemic shunt, Portal hypertension, Phase contrast MRI, Flow quantification, Flow visualization, Cardiovascular magnetic resonance

## Abstract

**Background:**

Four-dimensional (4D) flow cardiovascular magnetic resonance (CMR) is feasible for portal blood flow evaluation after placement of transjugular intrahepatic portosystemic shunts (TIPS) in patients with liver cirrhosis. However, clinical acceptance of 4D flow CMR in TIPS patients is limited due to the lack of validation studies. The purpose of this study was to validate 4D flow CMR-derived measurements in TIPS stent grafts using a three-dimensional (3D)-printed flow phantom.

**Methods:**

A translucent flow phantom of the portal vasculature was 3D-printed. The phantom consisted of the superior mesenteric vein and the splenic vein draining into the portal vein, the TIPS-tract, and the hepatic vein. A TIPS stent graft (Gore® Viatorr®) was positioned within the TIPS-tract. Superior mesenteric vein and splenic vein served as inlets for blood-mimicking fluid. 4D flow CMR acquisitions were performed at 3T at preset flow rates of 0.8 to 2.8 l/min using velocity encoding of both 1.0 and 2.0 m/s. Flow rates and velocities were measured at predefined levels in the portal vasculature and within the stent graft. Accuracy of 4D flow CMR was assessed through linear regression with reference measurements obtained by flow sensors and two-dimensional (2D) phase contrast (PC) CMR. Intra- and interobserver agreement were assessed through Bland–Altman analyses.

**Results:**

At a velocity encoding of 2.0 m/s, 4D flow CMR-derived flow rates and velocities showed an excellent correlation with preset flow rates and 2D PC CMR-derived flow velocities at all vascular levels and within the stent graft (all r ≥ 0.958, p ≤ 0.003). At a velocity encoding of 1.0 m/s, aliasing artifacts were present within the stent graft at flow rates ≥ 2.0 l/min. 4D flow CMR-derived measurements revealed high intra- and interobserver agreement.

**Conclusions:**

The in vitro accuracy and precision of 4D flow CMR is unaffected by the presence of TIPS stent grafts, suggesting that 4D flow CMR may be used to monitor TIPS patency in patients with liver cirrhosis.

**Supplementary Information:**

The online version contains supplementary material available at 10.1186/s12968-023-00920-5.

## Background

Portal hypertension is a potentially fatal complication of liver cirrhosis [1]. It is characterized by an increased portosystemic pressure gradient resulting in refractory ascites and gastroesophageal varices [2]. Implantation of a transjugular intrahepatic portosystemic shunt (TIPS) is an angiographic intervention for relieving the pressure in the portal system [3–5]. The TIPS stent graft diverts portal flow into the systemic circulation, thereby decreasing portal hypertension with subsequent reduction of ascites and risk of variceal bleeding [6, 7]. Possible complications of TIPS placement include excessive portosystemic shunting with the risk of TIPS-induced hepatic encephalopathy or TIPS dysfunction due to in-stent stenosis with recurrent portal hypertension [8]. For these reasons, comprehensive hemodynamic monitoring of the portal system and the TIPS stent graft is needed to monitor stent patency [9, 10].

Flow velocities are commonly assessed by Doppler ultrasonography (US) to monitor TIPS patency [3, 11]. However, Doppler US is limited by operator dependence and potentially reduced image quality due to gas-containing viscus or obesity [12, 13], precluding sensitive prediction of TIPS patency [14]. Therefore, a noninvasive, accurate, and operator-independent technique for comprehensive monitoring of portal blood flow and TIPS patency is desirable.

Volumetric blood flow is an alternative measure that can be used to assess TIPS function [15, 16]. Four-dimensional (4D) flow cardiovascular magnetic resonance (CMR) is a noninvasive technique that facilitates time-resolved, three-dimensional (3D) quantitative assessment of hepatic blood flow in a single acquisition with low interobserver variability and good reproducibility [17–19]. Previous pilot studies have shown the feasibility of 4D flow CMR for noninvasive monitoring of hepatic blood flow after TIPS placement in vivo [20–22]. Unfortunately, the potential of the TIPS stent graft mesh to induce artifacts and to influence the accuracy of 4D flow CMR-derived measurements has not been assessed due to the lack of appropriate reference standards. Adequate validation of 4D flow CMR-derived blood flow rates and flow velocities within TIPS stent grafts has not been performed. However, such validation is a prerequisite for further prospective studies and clinical acceptance of 4D flow CMR for monitoring of TIPS patency as well as for prediction of hepatic encephalopathy and refractory ascites after TIPS implantation.

Therefore, the purpose of our study was to validate 4D flow CMR-derived flow measurements in TIPS stent grafts using a 3D-printed flow phantom.

## Methods

### Flow phantom and experimental setup

**Fig. 1:**
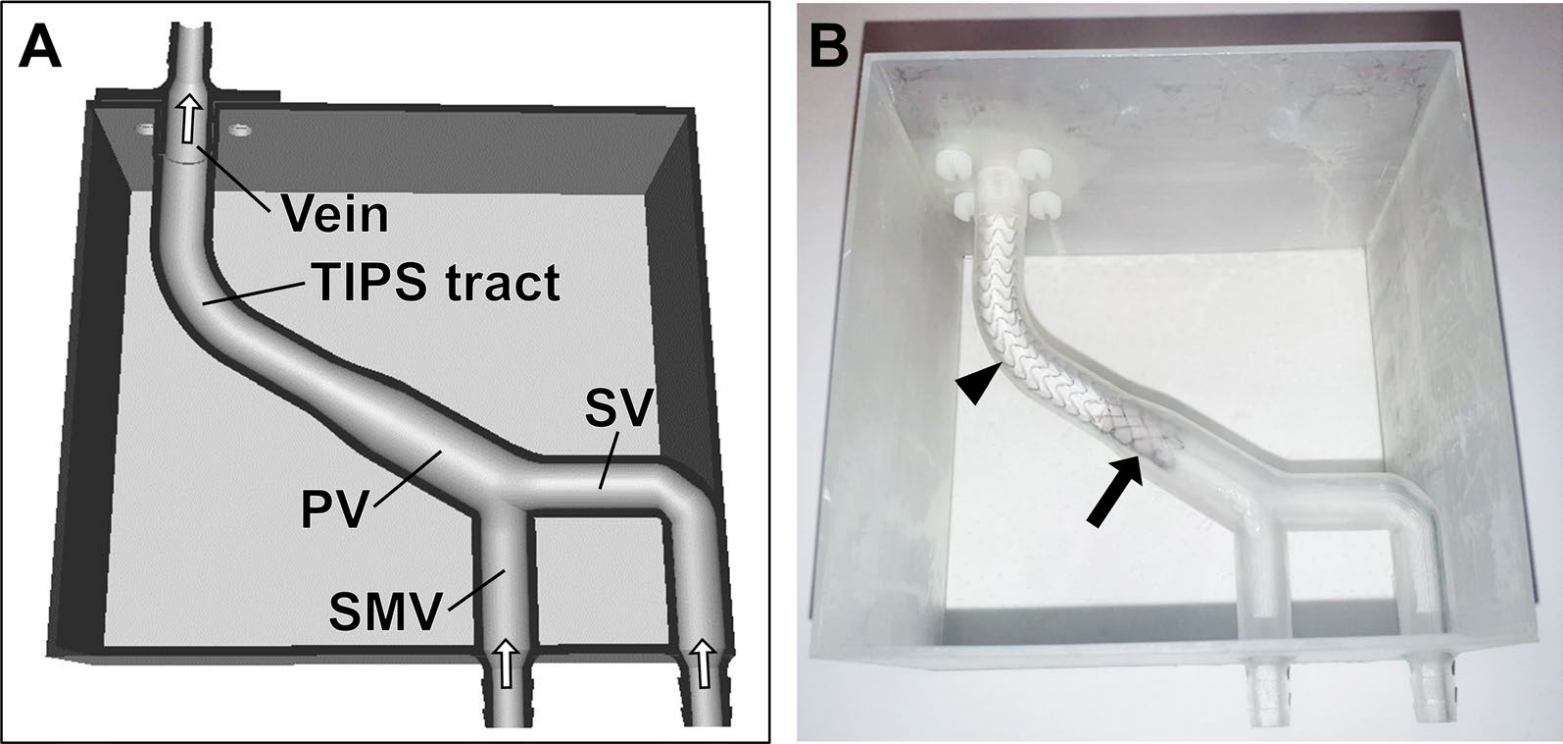
Flow phantom of the portal vasculature with transjugular intrahepatic portosystemic shunt (TIPS) stent graft. **A** Cross sectional rendering of the 3D phantom illustrating the design of the flow phantom mimicking the portal vasculature after TIPS implantation. The superior mesenteric vein (SMV) and the splenic vein (SV) serve as inlets. SMV and SV converge to the portal vein (PV), which narrows towards the TIPS tract. The TIPS tract drains into the hepatic vein (Vein) serving as outlet. White arrows indicate the flow direction. **B** Photograph of the translucent, 3D-printed phantom with the TIPS stent graft (Gore® Viatorr®) placed within the TIPS tract. The covered part of the TIPS stent graft is located within the TIPS tract (arrowhead). The bare part of the TIPS stent is located within the PV (arrow). The phantom was placed in the isocenter of a 3 T CMR system. The inlets of the phantom were connected via flexible tubes over two separate flow regulators and flow sensors to two pumps and the system was filled with blood-mimicking fluid

A flow phantom mimicking the portal vasculature was created with a 3D printer using Clear Resin (Form 3, Formlabs Inc., Somerville, Massachusetts, USA). The phantom consisted of the superior mesenteric vein (SMV, Ø10 mm) and the splenic vein (SV, Ø10 mm) draining into the extrahepatic portal vein (PV, Ø15 mm), the intrahepatic TIPS-tract, and the hepatic vein (Fig. 1A). Vessel diameters were chosen within the range of previously reported values [23–26].

A Viatorr® stent graft (W.L. Gore & Associates, Newark, Delaware, USA) was positioned within the TIPS-tract and PV. The stent graft had a diameter of 10 mm and measured 90 mm in length. The TIPS stent graft consisted of an expanded polytetrafluoroethylene covered part (70 mm) that was placed in the TIPS-tract and an uncovered part (20 mm) that was placed in the PV (Fig. 1B).

SMV and SV served as inlets and were connected via flexible tubes over two separate flow regulators and turbine flow sensors (FCH-midi-POM, B.I.O-TECH e.K., Vilshofen an der Donau, Germany) to two pumps. The system was filled with blood-mimicking fluid, consisting of a glycerol-water mixture with a volume fraction of glycerol of 0.331, resulting in a dynamic viscosity of 3.45∙10^–3^ Pa∙s at 20° C, similar to the viscosity of human blood at 37 °C [27, 28]. The reservoir around the vessels was filled with water to reduce susceptibility artifacts.

Flow rates were preset to 0.8, 1.2, 1.6, 2.0, 2.4, and 2.8 l/min and continuously monitored with the two flow sensors at the SMV and SV inlets.

### CMR imaging

CMR imaging was performed on a 3 T system (Ingenia, Philips Healthcare, Best, The Netherlands) with a 32-channel body phased array coil. An electrocardiogram (ECG) was simulated at a heart rate of 60 /min to perform ECG-triggered acquisitions for 4D flow CMR and two-dimensional (2D) phase contrast (PC) CMR.

The following cartesian 4D flow CMR acquisition parameters were applied: field of view, 240 × 320 × 187.5 mm^3^ (FH × AP × RL); acquired/reconstructed isotropic spatial resolution, 2.5 mm/1.25 mm; temporal resolution, 67 ms; repetition time/echo time, 3.5 ms/2.1 ms; flip angle, 4°; parallel imaging, SENSE (acceleration factor 4). Imaging time for each acquisition was 8 min 42 s. 4D flow CMR acquisitions covered the entire phantom.

The following 2D PC CMR acquisition parameters were applied: field of view, 300 × 300 mm^2^ (slice thickness 8 mm); acquired/reconstructed isotropic in-plane resolution, 1.5 mm/0.75 mm; temporal resolution 67 ms (15 time frames per cardiac cycle); repetition time/echo time, 4.5 ms/2.2 ms; flip angle, 10°. Imaging time for each 2D PC CMR acquisition was 23 s.

**Fig. 2:**
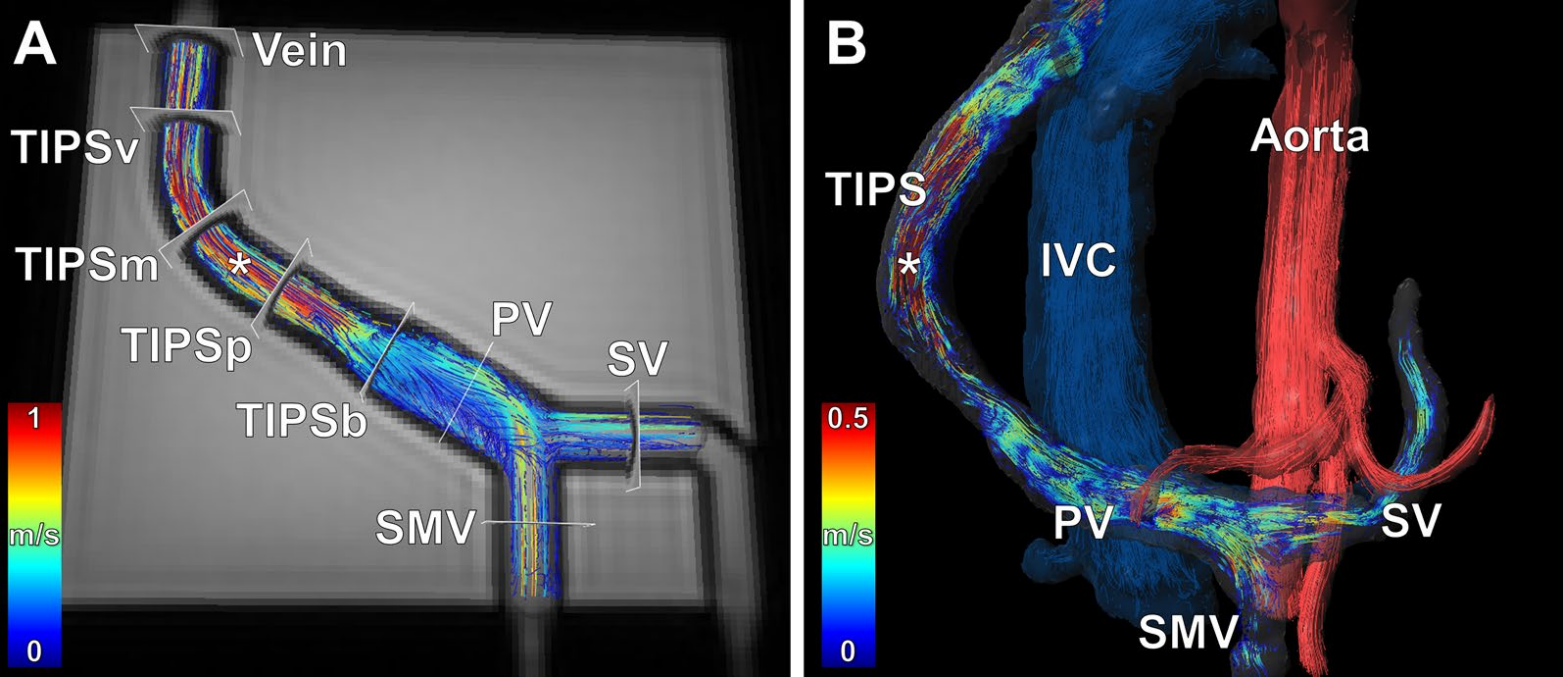
4D flow CMR of a TIPS stent graft within the flow phantom and within the human portal vasculature. **A** Velocity-coded 4D flow CMR image overlayed with a T2 weighted image of the flow phantom shows the velocity distribution in the portal circulation and TIPS stent graft, which is indicated by color-coded pathlines. Measurement levels for evaluation of flow rates and flow velocities are indicated: superior mesenteric vein (SMV), splenic vein (SV), portal vein (PV), bare part of the stent (TIPSb), each third of the covered stent (*TIPSp*  portal sided third of the stent, *TIPSm*  middle third of the stent, *TIPSv*  venous sided third of the stent), and vein. **B** 4D flow CMR imaging-based visualization of hemodynamics by color-coded pathlines in the portal system after TIPS placement in a 65-year-old woman. Blood flow is visualized within the aorta by red pathlines and within the inferior vena cava (IVC) by blue pathlines. Note the similarity of the velocity distribution in the flow phantom and the human portal vasculature after TIPS placement. Both the flow phantom and the patient reveal flow acceleration within the TIPS stent graft (asterisk)

2D PC CMR acquisitions were performed at the following predefined levels: SMV, SV, PV, the bare part of the stent (TIPSb), each third of the covered stent (TIPSp = portal sided third, TIPSm = middle third, TIPSv = venous sided third), and hepatic vein (Fig. 2). 4D flow CMR and 2D PC CMR were performed with velocity encoding of 1.0 m/s and 2.0 m/s at each preset flow rate.

### CMR data analysis

4D flow CMR datasets were automatically reconstructed to 15 time frames per cardiac cycle. Background phase offset correction was applied and 3D angiograms were rendered [29, 30].

One radiologist (CR) with three years of experience in 4D flow CMR assessment placed analysis planes at the above-mentioned levels: SMV, SV, PV, TIPSb, TIPSp, TIPSm, TIPSv, and the hepatic vein (Fig. 2). Flow rates and average through-plane velocities were quantified for each analysis plane. The software GTFlow 3.2.4 (GyroTools LLC, Zurich, Switzerland) was used for analysis of 4D flow CMR-derived datasets.

The same radiologist analyzed all 2D PC CMR datasets and quantified the average through-plane velocity using QFlow Analysis in IntelliSpace Portal 11.1 (Philips Healthcare).

### Intra- and interobserver agreement

4D flow CMR-derived flow rates and velocities within the covered stent were quantified three times at a velocity encoding of 2.0 m/s. For assessment of intraobserver agreement, two measurements were performed by the first radiologist (CR), with an interval of six months between the first and second measurement. For assessment of interobserver agreement, a third measurement was performed by a second reader (IR) with three years of experience in abdominal imaging.

### Statistical analysis

Preset flow rates monitored with flow sensors and 2D PC CMR-derived flow velocities served as standard of reference for 4D flow CMR-derived measurements. Measurements were excluded if velocities exceeded the preset velocity encoding and caused phase velocity aliasing artifacts. 4D flow CMR-derived parameters and standards of reference were compared using Bland–Altman analyses and Pearson’s correlation coefficient. A correlation was defined as strong if r > 0.8 and as excellent if r > 0.9 [31]. *P* < 0.05 indicated statistical significance. Bland–Altman analyses were performed to assess intra- and interobserver agreement of flow measurements within the stent graft. Data analysis was performed by Excel® (version 2201, Microsoft®, Redmond, Washington, USA) and SPSS  (version 20, Statistical Package for the Social Sciences, International Business Machines, Inc., Armonk, New York, USA).

## Results

**Fig. 3:**
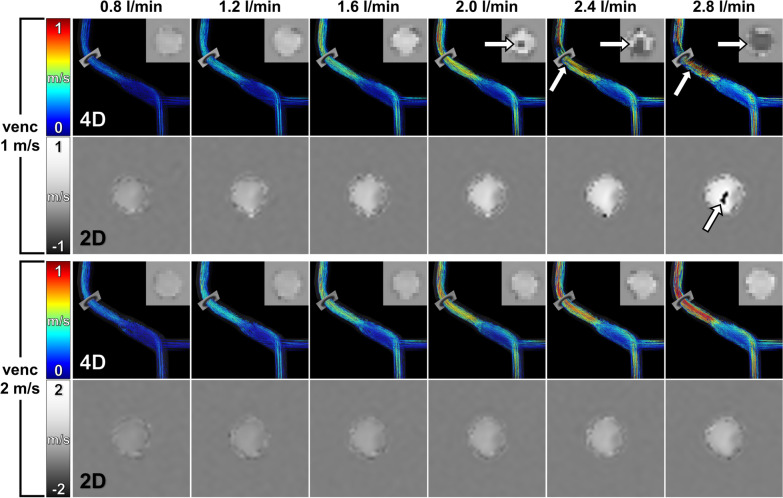
4D flow CMR and 2D PC CMR of the flow phantom at different flow rates. Preset flow rates were increased from 0.8 to 2.8 l/min (left to right). Comparative flow measurements were performed using velocity encoding both at 1 m/s (upper rows) and at 2 m/s (lower rows). Velocity-coded 4D flow CMR show velocity distribution in the portal system and TIPS stent graft. Color-coded pathlines demonstrate increasing flow velocities with increase of the preset flow rates. Measurement planes indicate the mid of the TIPS stent graft and correspond to the cross-sectional phase-contrast images shown for both 4D flow CMR (upper right corners) and 2D PC CMR (lower rows). Note that aliasing (arrows) occurred at higher flow rates and velocities when velocity encoding was set at 1 m/s. Aliasing was absent for all flow rates when velocity encoding set at 2 m/s

4D flow CMR was successfully performed at all preset flow rates of 0.8, 1.2, 1.6, 2.0, 2.4, and 2.8 l/min (Fig. 3).

**Fig. 4:**
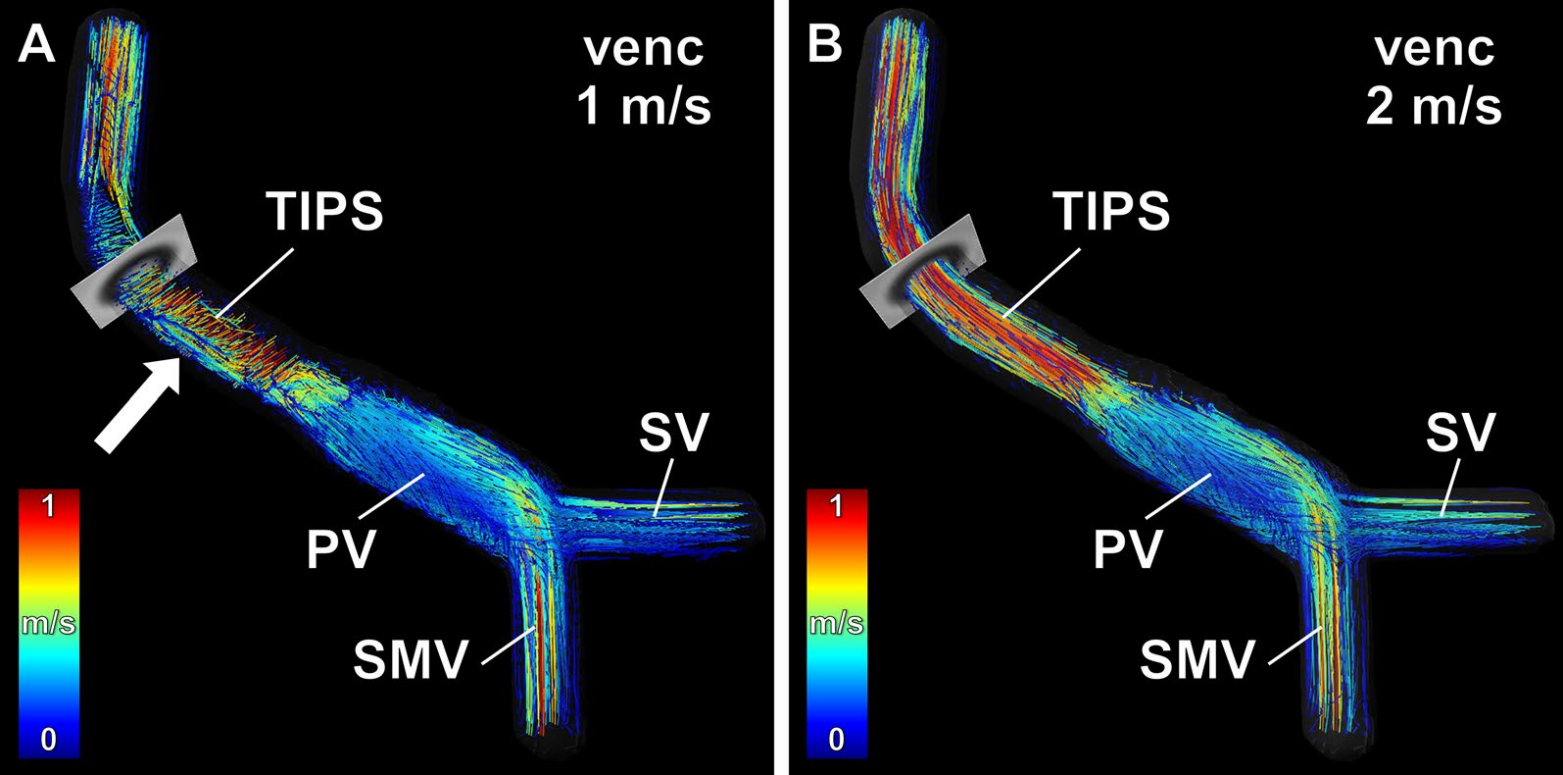
Comparison of low and high velocity encoded 4D flow CMR of the TIPS stent graft. 4D flow CMR performed at the maximum flow rate of 2.8 l/min resulted in flow acceleration within the TIPS stent graft above 1 m/s. **A** Velocity encoding at 1 m/s resulted in aliasing within the TIPS stent graft with consecutive erroneous calculation of pathlines as indicated by pathlines perpendicular to the fluid flow (arrow). **B** Velocity encoding at 2 m/s showed no aliasing within the TIPS stent graft and resulted in correct calculation of velocity-coded pathlines. Note that aliasing with erroneous calculation of pathlines results also in erroneous quantification of flow rates and flow velocities. *SMV*  superior mesenteric vein, *SV*  splenic vein, *PV*  portal vein

4D flow CMR-derived datasets with velocity encoding of 1.0 m/s could be analyzed completely at flow rates ≤ 1.6 l/min. At flow rates ≥ 2.0 l/min, aliasing occurred within the covered part of the stent graft and precluded 4D flow CMR-derived measurements of flow rates and flow velocities within the stent graft (Fig. 4A; Additional file 1). Eight measurements had to be excluded due to aliasing. With 2D PC CMR, aliasing occurred only at the highest flow rate of 2.8 l/min (Fig. 3).

At a velocity encoding of 2.0 m/s, 4D flow CMR-derived datasets and 2D PC CMR analysis planes could be completely analyzed at all preset flow rates (0.8 to 2.8 l/min) (Fig. 4B; Additional file 2; Additional file 3).

### Validation of 4D flow CMR-derived flow rates

**Fig. 5:**
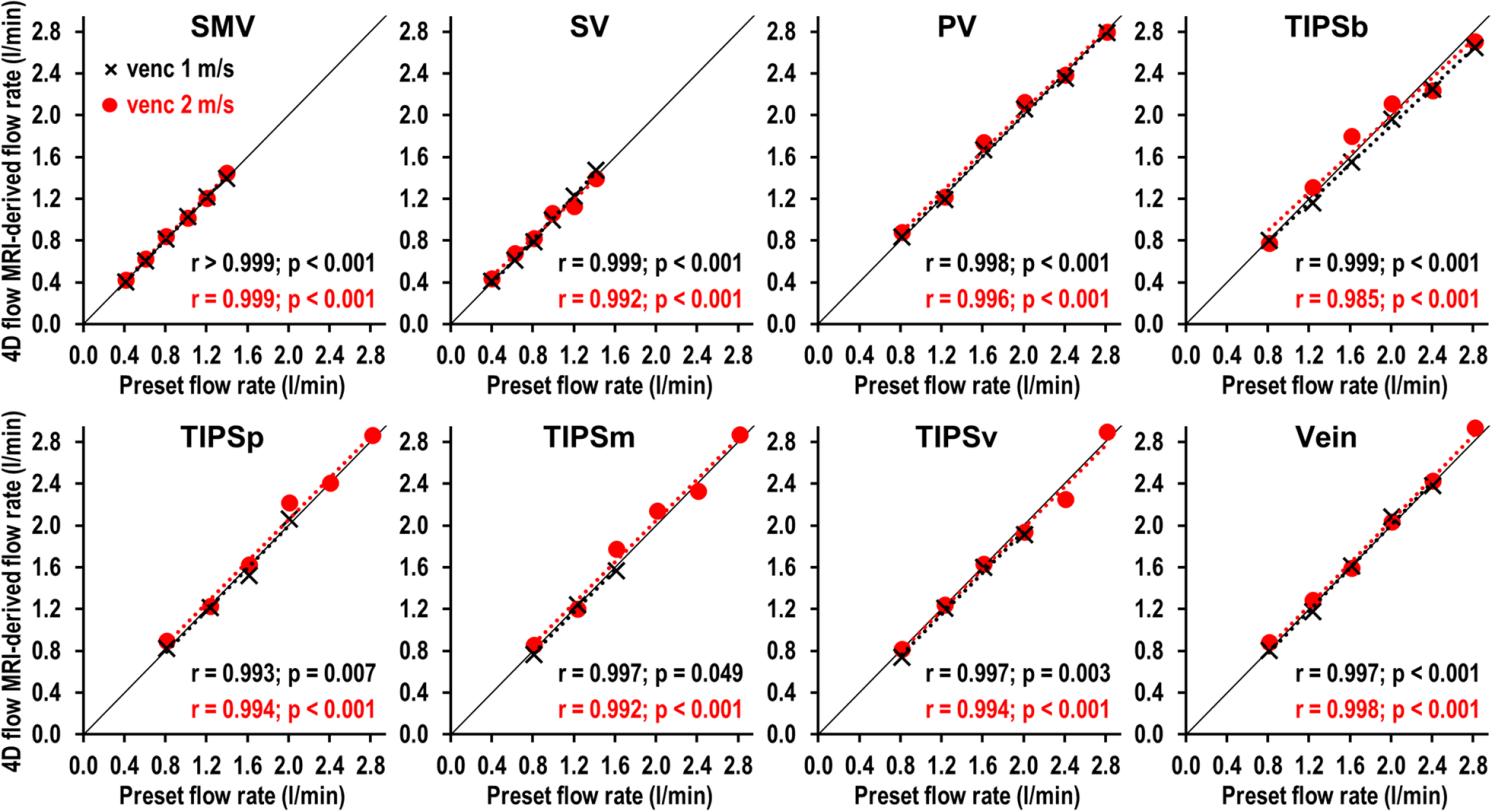
Correlation of 4D flow CMR-derived flow rates and preset flow rates. Preset TIPS flow rates were increased from 0.8 to 2.8 l/min and flow measurements were performed using velocity encoding both at 1 m/s (black crosses) and at 2 m/s (red dots). Flow measurements were performed at indicated levels. 4D flow CMR-derived flow rates were significantly correlated with the preset flow rates at all vascular levels and within the stent graft (all r ≥ 0.985 and all p ≤ 0.049). At velocity encoding of 1 m/s, flow measurements were excluded if velocities exceeded velocity encoding and caused aliasing artifacts. At velocity encoding of 2 m/s, aliasing did not occur. *SMV*  superior mesenteric vein, *SV*  splenic vein, *PV*  portal vein, *TIPSb*  bare part of the stent, *TIPSp*  portal sided third of the stent, *TIPSm*  middle third of the stent, *TIPSv*  venous sided third of the stent

4D flow CMR-derived flow rates at all vascular levels and within the stent graft showed excellent correlation with preset flow rates (0.8 to 2.8 l/min), which served as the standard of reference (Fig. 5).

At a velocity encoding of 1.0 m/s, the Pearson correlation coefficient was r ≥ 0.997 for flow rate measurements in the portal vessels (i.e., SMV, SV, PV) and the hepatic vein (all p < 0.001). The difference between the 4D flow CMR-derived flow rates and preset flow rates was 0.003 l/min (limits of agreement: − 0.063–0.070 l/min) in these vessels. Within the stent graft, the Pearson correlation coefficient was r ≥ 0.993 at all levels (i.e., TIPSb, p < 0.001; TIPSp, p = 0.007; TIPSm, p = 0.049; and TIPSv, p = 0.003). The difference between 4D flow CMR-derived flow rates and preset flow rates was − 0.051 l/min (limits of agreement: − 0.163–0.061 l/min) for in-stent measurements.

At a velocity encoding of 2.0 m/s, the Pearson correlation coefficient was r ≥ 0.992 for flow rate measurements in the portal vessels and the hepatic vein (all p < 0.001). The difference between the 4D flow CMR-derived flow rates and preset flow rates was 0.030 l/min (limits of agreement: − 0.068–0.128 l/min) in these vessels. Within the stent graft, the Pearson correlation coefficient was r ≥ 0.985 at all levels (all p < 0.001). The difference between 4D flow CMR-derived flow rates and preset flow rates was 0.023 l/min (limits of agreement: − 0.171–0.218 l/min) for in-stent measurements.

### Validation of 4D flow CMR-derived flow velocities

**Fig. 6:**
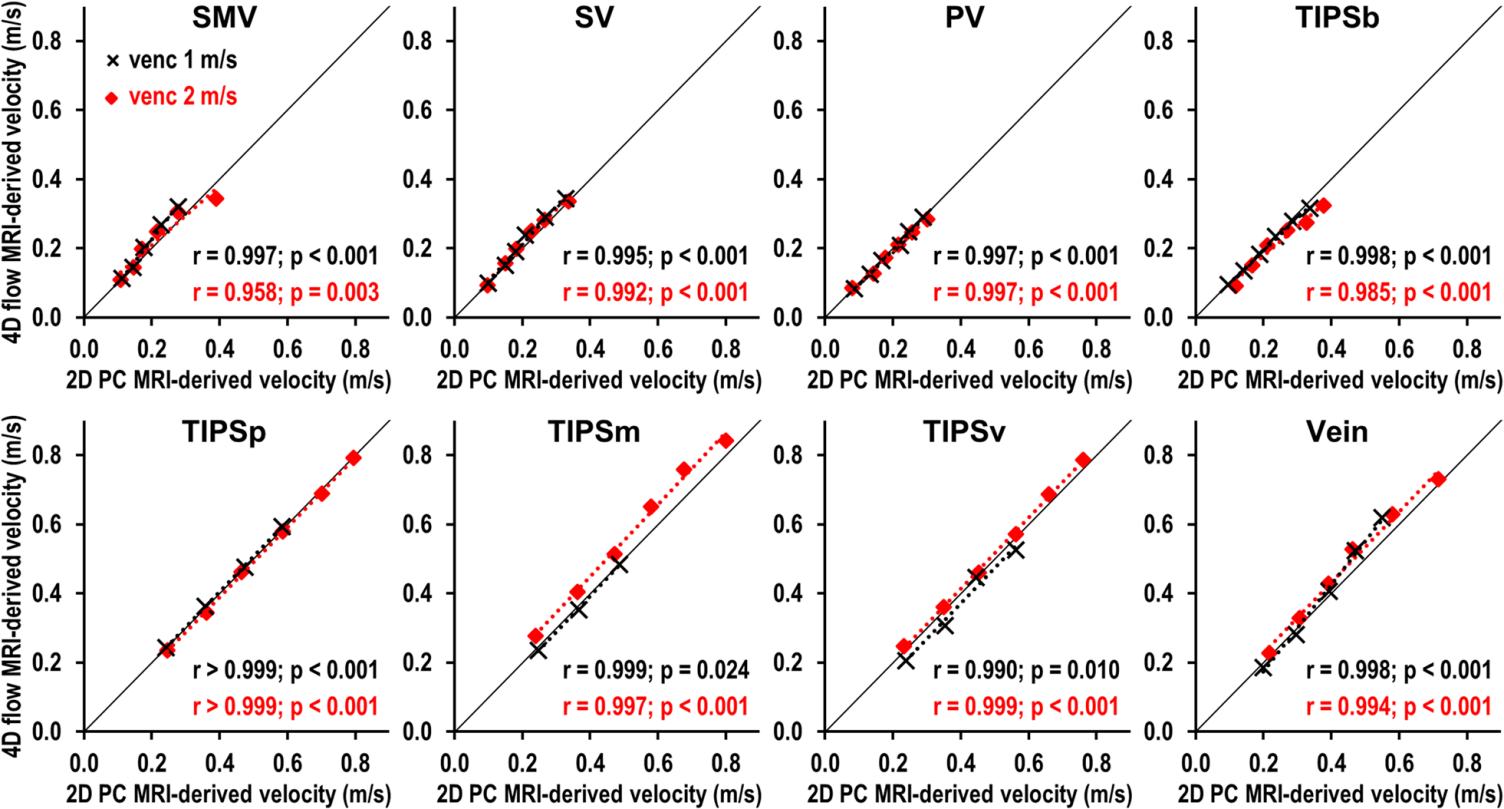
Correlation of 4D flow CMR- and 2D PC CMR-derived flow velocities. Preset flow rates were increased from 0.8 to 2.8 l/min and velocity measurements were performed using velocity encoding both at 1 m/s (black crosses) and at 2 m/s (red squares). Flow measurements were performed at indicated levels. 4D flow CMR-derived velocities were significantly correlated with 2D PC CMR-derived flow velocities at all vascular levels and within the stent graft (all r ≥ 0.958 and all p ≤ 0.024). At velocity encoding of 1 m/s, velocity measurements were excluded if velocities exceeded velocity encoding and caused aliasing artifacts. At velocity encoding of 2 m/s, aliasing did not occur. *SMV*  superior mesenteric vein, *SV*  splenic vein, *PV*  portal vein, *TIPSb*  bare part of the stent, *TIPSp*  portal sided third of the stent, *TIPSm*  middle third of TIPS stent, *TIPSv*  venous sided third of the stent

4D flow CMR-derived average flow velocities at all vascular levels and within the stent graft showed excellent correlation with 2D PC CMR-derived flow velocities, which served as the standard of reference (Fig. 6). 2D PC CMR-derived reference measurements ranged from flow velocities of 0.08 m/s in the PV and 0.23 m/s in the covered part of the stent at the minimal preset flow rate of 0.8 l/min to flow velocities of 0.30 m/s in the PV and 0.80 m/s in the covered part of the stent at the maximal preset flow rate of 2.8 l/min.

At a velocity encoding of 1.0 m/s, the Pearson correlation coefficient was r ≥ 0.995 for flow velocity measurements in the portal vessels (i.e., SMV, SV, PV) and the hepatic vein (all p < 0.001). The difference between the 4D flow CMR-derived flow velocity and the 2D PC CMR-derived flow velocity was 0.012 m/s (limits of agreement: − 0.032–0.057 m/s) in these vessels. Within the stent graft, the Pearson correlation coefficient was r ≥ 0.990 at all levels (all p ≤ 0.024). The difference between 4D flow CMR-derived flow velocity and 2D PC CMR-derived flow velocity was − 0.010 m/s (limits of agreement: − 0.041–0.022 m/s) for in-stent measurements.

At a velocity encoding of 2.0 m/s, the Pearson correlation coefficient was r ≥ 0.958 for flow velocity measurements in the portal vessels and the hepatic vein (all p ≤ 0.003). The difference between the 4D flow CMR-derived flow velocity and the 2D PC CMR-derived flow velocity was 0.010 m/s (limits of agreement: − 0.036–0.057 m/s) in these vessels. Within the stent graft, the Pearson correlation coefficient was r ≥ 0.985 at all levels (all p < 0.001). The difference between 4D flow CMR-derived flow velocity and 2D PC CMR-derived flow velocity was 0.008 m/s (limits of agreement: − 0.059–0.075 m/s) for in-stent measurements.

### Intra- and interobserver agreement

**Fig. 7:**
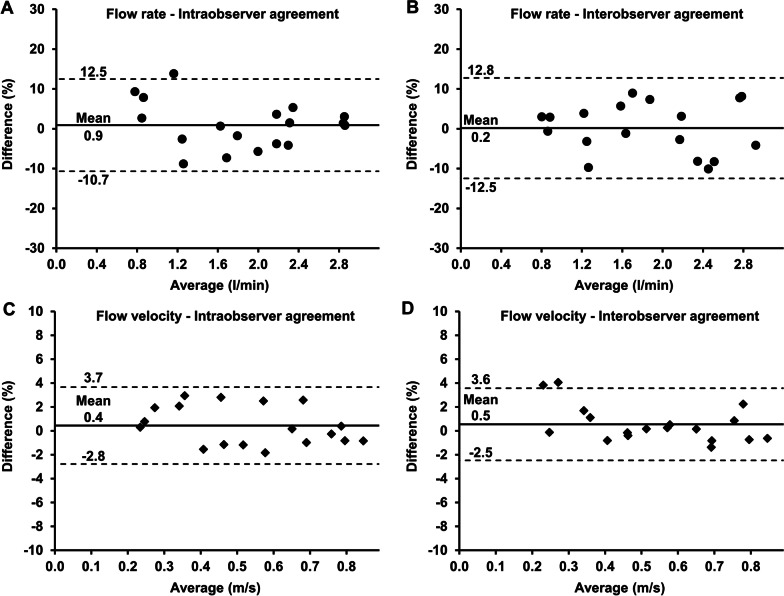
Intra- and interobserver agreement of 4D flow CMR-derived flow rate and velocity measurements. **A**, **B** Bland–Altman plots of intraobserver agreement (0.9 ± 5.9%) and interobserver agreement (0.2 ± 6.4%) for measurements of flow rates within the covered part of the TIPS stent graft (TIPSp, TIPSm, and TIPSv) at velocity encoding of 2 m/s. **C**, **D** Bland–Altman plots of intraobserver agreement (0.4 ± 1.6%) and interobserver agreement (0.5 ± 1.5%) for measurements of flow velocities within the covered part of the TIPS stent graft at velocity encoding of 2 m/s. Middle solid line indicates mean bias of flow rate and flow velocity measurements. Dashed lines indicate limits of agreement

Bland–Altman analyses of flow rate measurements within the TIPS stent graft revealed high intraobserver agreement (mean difference: 0.9%; limits of agreement: − 10.7–12.5%) as well as high interobserver agreement (mean difference: 0.2%; limits of agreement: − 12.5–12.8%) (Fig. 7A, B).

Bland–Altman analyses of flow velocity measurements within the TIPS stent graft revealed high intraobserver agreement (mean difference: 0.4%; limits of agreement: − 2.8–3.7%) as well as high interobserver agreement (mean difference: 0.5%; limits of agreement: − 2.5–3.6%) (Fig. 7C, D).

## Discussion

In this work we have successfully validated 4D flow CMR-derived flow measurements in TIPS stent grafts using a 3D-printed flow phantom. The use of the 3D-printed phantom facilitated rigorous evaluation of the accuracy of 4D flow CMR-derived flow rates and flow velocities in a model of the portal vasculature and also within a commonly used TIPS stent graft. Moreover, 4D flow CMR-derived in-stent measurements revealed high intra- and interobserver agreement.

Our phantom study was performed at flow rates ranging from 0.8 to 2.8 l/min. These in vitro flow rates in the TIPS stent graft were set to cover the typical range of 1.5 ± 1.0 l/min to 1.7 ± 0.5 l/min observed in previous in vivo pilot studies in TIPS patients [20, 21]. Therefore, our 4D flow CMR-based analyses using a 3D-printed flow phantom were performed under conditions that reflect portal flow rates that are commonly observed in patients with liver cirrhosis after TIPS placement.

The preset flow rates (0.8 to 2.8 l/min) resulted in average flow velocities ranging from 0.23 m/s to 0.80 m/s in the TIPS stent graft. These average flow velocities are not directly comparable to previous 4D flow CMR studies, reporting peak flow velocities in the range of 1.2 ± 0.2 to 1.3 ± 0.5 m/s [20, 21]. However, in the context of our validation study, we focused on measurement of average flow velocities, since peak velocity measurements are more sensitive to both noise and positioning of region of interest on analysis planes [32]. Further, peak velocities depend on the pulsatility of the flow that could not be modeled with our constant-flow phantom.

Our phantom study revealed high accuracy and high reliability of 4D flow CMR-derived flow rates and flow velocities within the TIPS stent graft as demonstrated by excellent correlation with reference standards (preset flow rates, 2D PC CMR) and high intra- and interobserver agreement. We are aware that in vitro estimations of accuracy and reliability using a 3D-printed phantom are overoptimistic compared to in vivo measurements, but nevertheless allowed for direct and rigorous validation of flow rates and velocities in TIPS stent grafts.

Comparative analyses of velocity encoding (1.0 m/s vs 2.0 m/s) revealed that peak flow velocities within the TIPS stent graft may exceed the velocity encoding at high flow rates if velocity encoding of 1.0 m/s was used. Consequently, aliasing artifacts occurred within the TIPS stent graft, precluding quantitative analyses. Aliasing artifacts were absent at a velocity encoding of 2.0 m/s. Thus, we recommend velocity encoding of 2.0 m/s for both qualitative and quantitative evaluation of TIPS hemodynamics in vivo, since flow might be further accelerated in patients with reduced lumen due to in-stent TIPS stenosis.

When comparing 4D flow CMR and 2D PC CMR, we observed aliasing at lower flow rates with 4D flow CMR as compared to 2D PC CMR. 2D PC CMR had a larger voxel size (1.5 × 1.5 × 8 = 18 mm^3^) compared to 4D flow CMR (2.5 × 2.5 × 2.5 mm^3^ = 15.6 mm^3^). The larger voxel size of 2D PC CMR leads to more intra-voxel flow averaging and might explain, at least in part, why 2D PC CMR was less prone to artifacts.

However, we believe that 4D flow CMR has several advantages compared to 2D PC CMR in patients with portal hypertension. 4D flow CMR does not only allow quantification of flow velocities and flow rates but also three-dimensional visualization of complex blood flow patterns in complex anatomical structures such as collaterals and varices [17]. Further, 4D flow CMR allows retrospective evaluation of these flow patterns off-line during post-processing [18].

Our phantom study has important potential scientific and clinical implications. Our in vitro validation may be helpful for future prospective in vivo studies. Further, this work adds confidence in 4D flow CMR-derived measurements needed for clinical acceptance of 4D flow CMR to monitor TIPS patency and to predict hepatic encephalopathy or refractory ascites after TIPS implantation.

### Limitations

Our phantom study has several limitations. First, our in vitro setting did not consider artifacts that frequently hamper 4D flow CMR examinations in vivo, such as breathing artifacts and other motion-related artifacts. Also, the signal to noise ratio of in vivo acquisitions may be lower, particularly in patients with TIPS dysfunction and refractory ascites. Regardless, 4D flow CMR-derived measurements in vitro provide important external validation to characterize the hemodynamics of TIPS stent grafts in the setting of portal hypertension.

Second, the 3D printed phantom represents only a simplification of the portal vasculature. All the fluid from the PV drained into the TIPS stent graft, since we did not model the right and left intrahepatic branch of the portal system. Our phantom therefore does not allow to gauge turbulence that may occur in vivo at the portal sided end of the TIPS stent graft, where the blood flow divides into the right PV and the TIPS stent graft.

Finally, we did not model in-stent stenoses of the TIPS. In-stent stenoses can cause flow acceleration requiring velocity encoding > 2.0 m/s. However, using velocity encoding > 2.0 m/s in vivo will result in increased noise [32] and potentially in erroneous blood flow quantification in portal vessels with slow flow velocities.

The application of dual-velocity encoded 4D flow CMR for improved assessment of high flow velocities in the TIPS stent graft and slow flow velocities in the portal vasculature might help address this potential issue [33]. Consequently, our next study using the flow phantom will compare the accuracy of a dual-velocity encoded 4D flow CMR to single velocity encoded 4D flow CMR acquisitions.

## Conclusions

The in vitro accuracy and precision of 4D flow CMR is unaffected by the presence of TIPS stent grafts, suggesting that 4D flow CMR may be used to monitor TIPS patency in patients with liver cirrhosis. Future prospective in vivo studies are warranted to determine the clinical role of 4D flow CMR, such as prediction of hepatic encephalopathy or refractory ascites after TIPS implantation.

## Supplementary Information

**Additional file 1: Movie S1.** Velocity-coded 4D flow CMR overlayed with a T2 weighted image of the flow phantom at a flow rate of 2.8 l/min using a velocity encoding of 1 m/s. Flow velocities within the covered part of the stent graft exceeded 1 m/s and resulted in aliasing artifacts with erroneous calculation of pathlines, precluding 4D flow CMR-derived measurements of flow rate and flow velocity.

**Additional file 2: Movie S2.** Velocity-coded 4D flow CMR overlayed with a T2 weighted image of the flow phantom at a flow rate of 2.8 l/min using a velocity encoding of 2 m/s. Flow velocities within the covered part of the stent graft did not exceed 2 m/s. Thus, the absence of aliasing allowed adequate 4D flow CMR-derived measurements of flow rate and flow velocity. Note the ability of 4D flow CMR for time-resolved visualization of complex blood flows such as mixing of the blood from the superior mesenteric vein and the splenic venin within the confluence venosum.

**Additional file 3: Movie S3.** Side-by-side comparison of 4D flow CMR-derived phase-contrast images within the portal vein and the TIPS stent using a velocity encoding of 1 m/s and 2 m/s at a flow rate of 2.8 l/min. At a velocity encoding of 1 m/s, aliasing occurred within the covered part of the TIPS stent graft while aliasing was absent within the portal vein. At a velocity encoding of 2 m/s, aliasing neither occurred within the portal vasculature nor the TIPS stent graft.

## Data Availability

The datasets used and/or analysed during the current study are available from the corresponding author on reasonable request.

## Abbreviations

2D: Two-dimensional
3D: Three-dimensional
4D: Four-dimensional
CMR: Cardiovascular magnetic resonance
ECG: Electrocardiogram
PC: Phase contrast
PV: Portal vein
SMV: Superior mesenteric vein
SV: Splenic vein
TIPS: Transjugular intrahepatic portosystemic shunt
TIPSb: Bare part of the TIPS stent
TIPSp: Portal sided third of the TIPS stent
TIPSm: Middle third of the TIPS stent
TIPSv: Venous sided third of the TIPS stent
US: Ultrasonography
